# The Acari Hypothesis, VI: human sebum and the cutaneous microbiome in allergy and in lipid homeostasis

**DOI:** 10.3389/falgy.2024.1478279

**Published:** 2024-11-21

**Authors:** Andrew C. Retzinger, Gregory S. Retzinger

**Affiliations:** ^1^ Department of Emergency Medicine, Camden Clark Medical Center, West Virginia University, Parkersburg, WV, United States; ^2^ Department of Pathology, Feinberg School of Medicine, Northwestern University, Chicago, IL, United States

**Keywords:** The Acari Hypothesis, allergy, sebaceous glands, sebum, *Malassezia*, *Staphylococcus aureus*, dyslipidemia, metabolic syndrome

## Abstract

The Acari Hypothesis posits that acarians, i.e., mites and ticks, are causative agents of IgE-mediated conditions. This report further develops The Hypothesis, providing rationale for the childhood predilection of allergy. In short, *Malassezia*, a fungus native to human skin and utterly dependent on sebaceous lipids, prevents allergy by deterring acarians. Because sebum output is limited before puberty, children are more prone to allergy than are adults. Competition for sebaceous lipids by *Staphylococcus aureus* influences not only *Malassezia* number—and, consequently, allergic predisposition—but also lipid homeostasis. The latter, in turn, contributes to dyslipidemia and associated conditions, e.g., the metabolic syndrome.

## 1 Introduction

Per the fourth installment of The Acari Hypothesis, modern hygienic practices disrupt human eccrine gland secretion, i.e., sweat, effectively increasing human—acarian interactions responsible for IgE-mediated allergic diseases (1). Although The Hypothesis and its corollaries provide rationales for why and how modern hygienic practices account for the ongoing allergy epidemic (2–5), the childhood predilection of some allergic diseases begs clarification. Indeed, the most remarkable epidemiologic finding shared by asthma, food allergy and atopic dermatitis is their increased incidence during childhood (6–8). Because IgE-mediated diseases are elicited by acarians acting on human epithelial surfaces, it is reasonable to assume epithelial surfaces of adults differ from those of children in a way that limits acarian activity. In this regard, the most conspicuous difference between the skin of pre- and post-pubertal humans is magnitude of sebum output by sebaceous glands (9, 10).

Sebum consists of a complex mixture of lipids that includes triglycerides, squalene, wax esters, cholesterol esters, free cholesterol, and fatty acids, Table 1 (11). Following puberty, sebum production increases 5-fold (9). Enhanced production continues through the seventh decade, after which the androgenic stimulation driving it decreases (9). Importantly, sebum output influences colonization of human skin by the lipid-dependent basidiomycete, *Malassezia*. Following puberty, *Malassezia* becomes the dominant eukaryote of the cutaneous microbiome (12), with malassezial colonization increasing by more than an order of magnitude (13, 14).

**Table 1:** Lipid composition of human sebum (11).

Lipid	% of Sebum Lipid
Triglycerides	30–50
Free Fatty Acids	15–30
Wax Esters	26–30
Squalene	12–20
Cholesterol Esters	3–6
Cholesterol	1.5–2.5

Because the behavior of *Malassezia* ranges from opportunism to commensalism to guardianship, the relationship between the fungus and humans has been difficult to characterize (12). The perception of *Malassezia* as opportunistic pathogen stems from its apparent etiologic involvement in many pathologies, most especially atopic dermatitis (AD) and seborrheic dermatitis (SD) (15–19). In the case of AD, afflicted persons synthesize IgE against an array of malassezial molecules (20, 21). According to The Hypothesis, the extensive targeting of such molecules by IgE indicates *Malassezia* is problematic for acarians (5). For this reason and because increased epithelial colonization by *Malassezia* aligns temporally with decreased incidence of allergic disease, the fungus participates in the anti-acarian defense of humans.

Although the hosting of fungi as a means of anti-acarian defense has never been described for mammals, mutualism of this sort has been described for plants. Indeed, some plants host fungal endophytes that protect them from bacterial and fungal pathogens and from phytophagous arthropods, including acarians (22). Fungal endophytes defend plants from acarians either via secretion of mycoacaricidal agents or via direct acarian inoculation (22).

Endophytic species of the basidiomycete, *Meira*, exemplify the acaropathogenic benefits fungal endophytes confer to host plants (23, 24). *Meira geulakonigii* protects citrus plants by killing the rust mite, *Phyllocoptruta oleivora* (25, 26). Culture medium from *M. geulakonigii* is acaricidal, indicating its anti-acarian activity is due to a toxin, not to fungal parasitism. As another example, *Meira argove* produces the mycoacaricide, argovin (4,5-dihydroxyindan-1-one), the anti-acarian effects of which have been well-characterized (27).

Given that other basidiomycetes protect their hosts from acarian parasitism, it is entirely reasonable to assume *Malassezia* protects humans from similar fate. Positioning *Malassezia* within sebaceous glands that constitutively secrete sebum provides a convenient means to deliver a *Malassezia*-derived anti-acarian agent to an epidermal surface. If humans provide to the fungus an essential nutrient whilst the fungus protects humans from acarian infestation, then the relationship between humans and *Malessezia* is most appropriately considered mutualistic. Such mutualism surely influences the physiology and pathophysiology of humans in unappreciated ways.

As for the involvement of *Malassezia* in human disease, firstly, not all *Malassezia* are constituents of normal skin flora, with some cross-colonization occurring due to cohabitation of humans and domestic mammals (28). Secondly, human sweat creates a microenvironmental ecosystem unfavorable not only to acarians, but also to a multitude of other microorganisms (29). Thus, even as disruption of the ecosystem by, for example, hygienic measures, enables problematic encounters between acarians and humans, it also enables problematic encounters between other invasive microorganisms and native *Malassezia*. As will be discussed next, *Malassezia*-associated diseases of humans are consequences of competition between native epidermal *Malassezia* and invasive organisms, e.g., *Staphylococcus aureus*.

## 2 Malassezia and AD

*Malassezia* is a lipophilic basidiomycete that inhabits epithelia of warm-blooded animals (30). The fungal genus is the only one included in class Malasseziomycetes, subphylum Ustilaginomycotina, a taxon consisting primarily of plant pathogens (31). To date, 18 species of *Malassezia* have been identified (32).

Despite a requirement for long chain fatty acids, *Malassezia* lacks a gene for fatty acid synthase (33), rendering the fungus the only free-living one not able to synthesize fatty acids (34). Consequently, *Malassezia* must exploit exogenous lipid sources to survive. Human epidermis, upon which is constitutively secreted an abundance of fatty acids (35), is an ideal environment for malassezial colonization and propagation.

Although *Malassezia* subsists on the lipid-rich secretions of mammalian sebaceous glands, the relationship between mammals and *Malassezia* has, to date, not been considered mutualistic because: (1) colonization by the fungus has not been appreciated to confer substantial benefit to mammalian hosts, and (2) the fungus seems to play a role in some human diseases (12). As an important example of the latter, individuals with AD express anti-malassezial IgE (20, 21), which may influence the symptoms of the disorder (4).

Pathogen recognition receptors (PRRs) are utilized by invertebrates and vertebrates to identify and neutralize deleterious materials. As an example of invertebrate usage, ticks secrete into their saliva immunoglobulin-binding proteins that adsorb to and neutralize immunoglobulins ingested during a blood meal (36). Per The Hypothesis, mammals exploit acarian PRRs in the formation of IgE, to protect themselves from acarian vectorial activity (3). The anti-acarian specificity of IgE follows from how allergenicity is borne and conveyed. Namely, following complexation with an acarian PRR within the acarian digestive tract, substances become targeted by IgE when inoculated into a human, e.g., during an invasive encounter (3). IgE-targeted materials derive from either the acarian or its foodstuffs. The targeting of malassezial molecules by IgE indicates those molecules contributed to the diet of the operative acarian (5).

The ubiquity of acarians ensures frequent encounters with humans. Examples include well-defined ectoparasitism by *Sarcoptes scabiei*, *Demodex* spp. and a multitude of tick species. More subtle encounters involve synanthropic *Pyroglyphidae*, e.g., *Dermatophagoides pteronyssinus* and *Dermatophagoides farinae*. Indeed, these house dust mites may contribute most to the development of allergy: (1) they exist in increased number on the skin and in the homes of patients with IgE-mediated disease (37, 38), and (2) they are intimately associated with sources of common allergens. With regard to this last, note especially that house dust mites consume human and pet dander, fungi and wheat, and they use cockroaches as phoretic hosts (39–42).

Given that the primary foodstuffs of dust mites are human epidermal materials, the digestive tract of dust mites must be exposed to malassezial elements routinely (43). Furthermore, because most skin flora are not targeted by IgE, the specificity of the antibody for malassezial materials indicates a special affinity of the acarian PRR for *Malassezia*. For this reason, and because the increase in malassezial colonization that follows puberty coincides with the decrease in the incidence of allergy, it is reasonable to assume *Malassezia* is involved in the anti-acarian defense of humans.

Humans are not the only animals with sebaceous glands. Indeed, all extant mammalian lineages either now express sebaceous glands or did in the past (44–46). Like humans, many of the other mammals that have sebaceous glands host *Malassezia* (47–49). Because those mammals, too, are subject to acarian parasitism, sebaceous glands somehow effectively ward off acarians. Given that IgE and sebaceous glands are both defining features of mammalian species and cardinal to mammalian anti-acarian defense, it appears acarians very significantly influenced mammalian evolution. If that is the case, then other mammalian adaptations also arose in response to evolutionary pressure exerted by acarians.

Mammary glands may predate the origin of class Mammalia, but their modern-day expression is limited to extant mammalian lineages (50). Although the evolutionary pressure responsible for emergence of mammary glands is poorly understood, a leading theory holds that the glands evolved as a neomorphic mosaic, combining the properties of both sebaceous and apocrine glands (51). If a primary function of sebaceous glands is support of epidermal colonization by *Malassezia*, then mammary glands must somehow promote the growth and vertical transmission of the fungus. It should come as no surprise that *Malassezia* represents the dominant fungus present in the breastmilk of healthy mothers (52). Indeed, transmission of *Malassezia* between human mothers and their progeny has already been demonstrated: 89% of infants have detectable levels of dermal *Malassezia* on day 0, with 100% having detectable levels by day 1 (53). By day 30, malassezial diversity conforms to that of adults, with fungal genotypes being those of mothers (53). As will next be argued, not only does mutualism between humans and *Malassezia* provide rationale for mammary glands, but it also provides insight into human dermatopathologies attributed to *Malassezia*.

## 3 Adaptations and associations

The Acari Hypothesis clarifies the role of some human adaptations in the anti-acarian defense of humans. According to The Hypothesis, both endogenous molecules, e.g., dermcidin and apolipoprotein D, and skin microbiota, e.g., *Malassezia*, protect humans from the vectorial threat posed by acarians (5). Although The Hypothesis was developed primarily to help ‘decipher’ allergy, it also addresses other matters pertinent to human pathophysiology, especially ones relating certain dermatopathologies to dyslipidemia (54, 55).

Multiple dermatopathologies are associated with dyslipidemia, including, most notably, SD, a chronic recurring skin condition characterized by greasy erythematous plaques and yellow-gray scale (56). Because anti-fungal therapy resolves the symptoms of SD, *Malassezia* is believed central to the pathophysiology of the disorder (57, 58). Relatedly, *Malassezia* influences sebum content via metabolism of triglycerides and liberation of free fatty acids, especially oleic acid (59). For reasons yet unknown, skin of persons with SD reacts strongly to oleic acid whilst skin of healthy individuals does not (60).

Although *Malassezia* is believed critical to the etiology of SD, the skin of persons with the disorder is also characterized by a striking bacterial dysbiosis, with *S. aureus* being the most abundant microorganism (61). *S. aureus* is a facultative, anaerobic, gram-positive bacterium that has historically been considered a constituent of the normal flora of human skin and nasal passages (62, 63). In addition to being an opportunistic pathogen, *S. aureus* contributes to the pathophysiology of IgE-mediated diseases, including AD, allergic rhinitis, and asthma (64–66). Like *Malassezia*, *S. aureus* is unusual in that persons with allergic conditions often produce IgE against proteins expressed by the organism. One study found that 27% of dust mite-sensitized patients who suffer from both asthma and allergic rhinitis express IgE against toxic shock syndrome toxin-1 (TSST-1) (66), a protein secreted by *S. aureus*. Another study found that 38% of patients with moderate AD express IgE against TSST-1 (67). As follows from The Hypothesis, the existence of IgE against TSST-1 confirms interaction between *S. aureus* and acarians and suggests TSST-1 has anti-acarian activity.

Numerous studies have shown oleic acid significantly impacts *S. aureus* physiology by decreasing bacterial adhesion (68), disrupting cell membranes (69) and/or limiting expression of bacterial virulence genes (70). Inasmuch as *S. aureus* is the most abundant organism on the skin of individuals with SD, it is entirely possible oleic acid reactivity is not a direct response to the lipid, rather it is a secondary response to materials expressed by *S. aureus*.

Importantly, molecular constituents of eccrine gland secretions have antimicrobial activity against *S. aureus* (29, 71). Just as hygienic removal of eccrine gland secretions fosters acarian infestation, it also fosters *S. aureus* colonization. Consequently, just as acarian dysbiosis can cause human disease, i.e., allergy, so, too, can bacterial dysbiosis cause human disease, e.g., SD. If the relationship between *Malassezia* and humans is mutualistic, then *Malassezia* must be native to the human epidermal ecosystem/microbiome. It follows that *S. aureus* should not be considered normal skin flora. Instead, the bacterium should be considered a strict pathogen; one that is invasive to the epidermal ecosystem and only present on contemporary humans because of modern hygiene.

Unlike *Malassezia*, *S. aureus* does not depend on exogenous fatty acids for its survival (72). Still, because *de novo* synthesis of fatty acids for bacterial membrane inclusion requires substantial energy expenditure, *S. aureus* scavenges fatty acids of its host (73, 74). Consistent with this operation, *S. aureus* expresses lipases that liberate fatty acids from triglycerides, the major component of sebaceous gland secretions (75). The importance of sebaceous lipids to *S. aureus* is further supported by the microanatomic distribution of the bacterium: its colonies preferentially cluster around pilosebaceous units (76, 77). Given both the lipid dependence of *Malassezia* and the co-localization of *Malassezia* and *S. aureus*, the two organisms undoubtedly compete for host lipids. The response of *Malassezia* toward *S. aureus* may be one of self-preservation. Alternatively, the anti-staphylococcal activity of *Malassezia* may benefit the host directly, a consequence of unappreciated evolutionary design.

Evidence indicates epidermal competition between *Malassezia* and *S. aureus* has systemic consequences. As one instructive example, severe SD is associated with development and progression of the dyslipidemia characteristic of the metabolic syndrome (MetS) (78, 79). If *Malassezia* contributes to the antimicrobial defense of humans, and sebum enables epidermal colonization by *Malassezia*, then the fungus influences lipid homeostasis. Further, because systemic lipids are undoubtedly trafficked to sebaceous glands during the epidermal inflammatory response, epidermal co-localization of pathogens that influence the well-being of either *Malassezia* or its human host may beget dyslipidemia.

## 4 Closing

The Acari Hypothesis is a construct with which to address unknowns relevant to IgE-mediated disease. As with any disease-related hypothesis, its utility depends upon its ability to facilitate mechanistic understanding. In this regard, not only does The Hypothesis provide rationale satisfying to allergy and its related issues, but it also helps to answer questions relevant both to human evolution and to pathophysiologies of other diseases. As one very salient example of the latter, MetS refers to co-occurrences of insulin resistance, obesity, dyslipidemia and hypertension (80). Persons who have MetS are at elevated risk of cardiovascular disease (81). As elaborated above, dysbiosis that results in *S. aureus* colonization and subsequent competition with *Malassezia* may yield the dyslipidemia of MetS. Indeed, in some animal models, *S. aureus* infection induces both insulin resistance and adipogenesis (82–84). Thus, *S. aureus* may be the causative agent of MetS. If that is so, then hygienic practices may drive heart disease in developed countries (ACR, submitted).

## Data Availability

The original contributions presented in the study are included in the article/Supplementary Material, further inquiries can be directed to the corresponding author.
